# Multisystem inflammatory syndrome associated with SARS-CoV-2 infection in children: update and new insights from the second report of an Iranian referral hospital

**DOI:** 10.1017/S0950268822001522

**Published:** 2022-10-18

**Authors:** Setareh Mamishi, Mehrnaz Olfat, Babak Pourakbari, Hamid Eshaghi, Mohammad Reza Abdolsalehi, Mohammad Ali Shahbabaie, Fatemeh Jalali, Fatemeh Safari, Shima Mahmoudi

**Affiliations:** 1 Pediatric Infectious Disease Research Center, Tehran University of Medical Sciences, Tehran, Iran; 2 Department of Infectious Diseases, Pediatrics Center of Excellence, Children's Medical Center, Tehran University of Medical Sciences, Tehran, Iran; 3 Pediatrics Center of Excellence, Children's Medical Center, Tehran University of Medical Sciences, Tehran, Iran

**Keywords:** COVID-19, MIS-C, SARS-CoV-2

## Abstract

**Introduction:**

Here, we are sharing our second report about children affected by Multisystem Inflammatory Syndrome in Children (MIS-C). The aim of the present study was to update our knowledge about children with MIS-C. Furthermore, we tried to compare clinical manifestations, laboratory features and final outcome of patients based on disease severity, in order to better understanding of the nature of this novel syndrome.

**Methods:**

This retrospective study was conducted at Children's Medical Center Hospital, the hub of excellence in paediatrics in Iran, located in Tehran, Iran. We reviewed medical records of children admitted to the hospital with the diagnosis of MIS-C from July 2020 to October 2021.

**Results:**

One hundred and twenty-two patients enrolled the study. Ninety-seven (79.5%) patients had mild to moderate MIS-C (MIS-C without overlap with KD (*n* = 80); MIS-C overlapping with KD (*n* = 17)) and 25 (20.5%) patients showed severe MIS-C. The mean age of all patients was 6.4 ± 4.0 years. Nausea and vomiting (53.3%), skin rash (49.6%), abdominal pain (46.7%) and conjunctivitis (41.8%) were also frequently seen Headache, chest pain, tachypnea and respiratory distress were significantly more common in patients with severe MIS-C (*P* < 0.0001, *P* = 0.021, *P* < 0.0001 and *P* < 0.0001, respectively). Positive anti-N severe acute respiratory syndrome coronavirus 2 IgM and IgG were detected in 14 (33.3%) and 23 (46.9%) tested patients, respectively. Albumin, and vitamin D levels in children with severe MISC were significantly lower than children with mild to moderate MIS-C (*P* < 0.0001, *P* = 0.05). Unfortunately, 2 (1.6%) of 122 patients died and both had severe MIS-C.

**Conclusion:**

Patients with MIS-C in our region suffer from wide range of signs and symptoms. Among laboratory parameters, hypoalbuminemia and low vitamin D levels may predict a more severe course of the disease. Coronary artery dilation is frequently seen among all patients, regardless of disease severity.

## Introduction

Coronavirus disease 2019 (COVID-19) caused by severe acute respiratory syndrome coronavirus 2 (SARS-CoV-2) was first detected in December 2019, in Wuhan, China and rapidly spread throughout the world [1]. World Health Organization (WHO) introduced COVID-19 as a pandemic on March 2020.

COVID-19 disease results in spectrum of signs and symptoms, ranging from asymptomatic to severe pneumonia and death [2, 3]. Children are also affected by COVID-19, but they have less severe disease and better outcome than adults [2, 4].

On spring 2020, authors announce that a remarkable increase in Kawasaki disease (KD)-like syndrome outbreak in children, is happening, which results in a multisystem inflammatory phenomenon, with footprint of COVID-19 [5–8]. They called this new entity: Multisystem Inflammatory Syndrome in Children (MIS-C). The signs and symptoms of this cytokine mediated syndrome are fever and multiple organ involvement, which results in critically illness requiring hospitalisation and subsequent morbidities in many of cases [9]. Among them, cardiac involvements, including coronary arteries dilation are of particular importance [10, 11]. The association with COVID-19 can be demonstrated by positive SARS-CoV-2 RT-PCR or more common positive SARS-CoV-2 serology; and even a history of exposure to a COVID-19 patient [12]. Many authors studied the demographic features, clinical manifestation and therapeutic approaches of this syndrome [13–15]. We also published our first experience in 45 children affected by MIS-C on August 2020 [16]. Here, we are sharing our second report about children affected by MIS-C. The aim of the present study was to update our knowledge about children with MIS-C in our region. Furthermore, we tried to compare clinical manifestations, laboratory features and final outcome of patients based on disease severity, in order to better understanding of the nature of this novel syndrome.

## Materials and methods

The Research deputy and Ethics committee of Tehran University of Medical Sciences approved the study (IR.TUMS.VCR.REC.1399.057). Since the study was done retrospectively, no further intervention was applied and all of the diagnostic and therapeutic measures were performed based on physician's preference in the duration of hospitalisation; nonetheless, we obtaine a general written informed consent from parents of all patients at the time of admission.

This retrospective study was conducted at Children's Medical Center Hospital, the hub of excellence in paediatrics in Iran, located in Tehran, Iran. All patients with suspected MIS-C have paediatric cardiology, paediatric infectious disease and paediatric rheumatology consults. Patients were evaluated for demographic characteristics, history of contact with confirmed or suspected cases of COVID-19, presenting symptoms, clinical and laboratory features, cardiac involvement, treatment modalities and outcomes. The echocardiography (ECHO) and ECG findings for the patients were recorded at the time of admission with follow-up within 2 weeks post discharge with paediatric cardiology.

Renal involvement was defined according to the kidney function, serum Blood Urea Nitrogen, creatinine sodium, potassium, calcium and glomerular filtration rate according to Schwartz formula.

We reviewed medical records of children admitted to the hospital with the diagnosis of MIS-C from July 2020 to October 2021. We have published our first experience in children with MIS-C on August 2020 [16].

## Case definition

Several MIS-C case definitions have been described by national and international committees. We use the CDC's criteria to diagnose the disease in our centre [17]. Characteristics principles for MISC diagnosis as follows:

1. Age under 21 years
2. Fever lasting 24 h or more (documented or subjective)
3. Presence of inflammatory markers (increased ESR, CRP, fibrinogen, procalcitonin, D-dimer, ferritin, LDH, IL-6 and neutrophil; as well as lymphocytopenia and hypoalbuminemia)
4. At least two organ involvement (cardiovascular, respiratory, renal, neurologic, haematologic, gastrointestinal and dermatologic)
5. Severe illness requiring hospital admission
6. No other probable diagnosis
7. Footprint of SARS-CoV-2 infection or exposure (positive SARS-CoV-2 RT-PCR, positive SARS-CoV-2 serology, positive SARS-CoV-2 antigen test and exposure to a patient with COVID-19 within last four weeks)

We also studied patients in two distinct groups (mild/moderate, and severe) based on the Vasoactive-Inotropic Score (VIS), degree of respiratory support and evidence of organ injury [18] (Table 1).

**Table 1.** Disease severity assessment in children diagnosed with MIS-C

Variables	Disease severity		
	Mild	Moderate	Severe
Supplemental oxygen /respiratory support	No	Mask or nasal canula	High flow oxygen and/or mechanical ventilation
Vasoactive drugs requirement	No	Yes	Yes
Organs involvement	Minor	Minor or limited Or coronary arteries involvement	Moderate to severe organ involvement such as ventricular dysfunction and coronary artery aneurysm
Progressive disease	No	Yes	Yes

Although distinguishing patients with KD-like MIS-C from those with true KD is usually difficult, the classification of MIS-C *vs.* KD is based on SARS-CoV-2 testing and exposure history. Patients with positive SARS-CoV-2 testing (or with an exposure to an individual with COVID-19) who also fulfil criteria for complete or incomplete KD are considered to have MIS-C overlapping with KD.

## Therapeutic team

Our main therapeutic team consisted of general paediatrician, paediatric infectious disease specialist, paediatric rheumatologist, paediatric intensivist, paediatric cardiologist and pharmacologist. As this hospital is a tertiary referral hospital with all subspecialist available, we consulted with other physicians when needed.

## Data analysis

The statistical analyses were performed using Statistical Package for the Social Sciences (SPSS) version 13.0 software (SPSS Inc.). Categorical variables were reported as frequency and percentages. Normally distributed continuous variables were presented as means with standard deviations (s.d.) and not normally distributed variables were described using median and interquartile range (IQR) values. For comparing laboratory parameters between the groups, the nonparametric tests (Mann–Whitney) or parametric tests (non-paired Student t test) were performed. *P* < 0.05 was considered as statistically significant.

## Results

One hundred and twenty-two patients enrolled the study. Ninety-seven (79.5%) patients had mild to moderate MIS-C (MIS-C without overlap with KD (*n* = 80); MIS-C overlapping with KD (*n* = 17)) and 25 (20.5%) patients showed severe MIS-C.

Forty-eight (39.3%) patients were female and 74 (60.7%) patients were male. There was no significant difference in sex distribution between three groups (*P* = 0.837). The mean age of all patients was 6.4 ± 4.0 years. The mean age of patients with severe MIS-C (8.2 ± 4.6 years) was significantly higher than MIS-C group without overlap with KD (5.7 ± 3.7 years) and MIS-C group overlapping with KD (6.75 ± 4.4 years), respectively (*P* = 0.029). Fifteen (12.3%) patients had underlying disease; 11 (11.3%) patients with mild and moderate MIS-C and 4 (16%) patients with severe MIS-C. This was not a noticeable difference (*P* = 0.507).

Totally, 74 patients (60.7%) had the history of close contact with an individual with illness clinically compatible with COVID-19 disease who had close contact with an individual with laboratory-confirmed SARS-CoV-2 infection; however, there was no significant difference between two groups (*P* = 0.172). This was found in 62 cases with mild and moderate MIS-C (64%).

We also studied serologic tests as a footprint of COVID-19 in some of our patients as indicated. Positive anti-N SARS-CoV-2 IgM and IgG were detected in 14 (33.3%) and 23 (46.9%) tested patients, respectively. There was no significant difference between mild to moderate and severe disease (*P* = 1.000 and *P* = 0.734 respectively). We also obtained a nasopharyngeal SARS-CoV-2 RT-PCR test for all of the patients: 36.9% of samples were positive; 37.1% in patients with mild and moderate disease and 36.9% in patients with severe disease (*P* = 1.000).

The most common clinical manifestation at the presentation was fever which was documented in all patients. Nausea and vomiting (53.3%), skin rash (49.6%), abdominal pain (46.7%) and conjunctivitis (41.8%) were also frequently seen. We also compared clinical manifestations between three groups (MIS-C overlapping with KD, MIS-C without overlap with KD and severe MIS-C). Headache, chest pain, tachypnea and respiratory distress were significantly more common in patients with severe MIS-C (*P* < 0.0001, *P* = 0.021, *P* < 0.0001 and *P* < 0.0001, respectively). Table 2 shows the clinical manifestations in detail.

**Table 2.** Clinical characteristics of patients with MIS-C in different groups at presentation

Variables *N* (%)	Disease severity				
	Total (*n* = 122)	MIS-C overlapping with KD (*n* = 17)	MIS-C without overlap with KD (*n* = 80)	Severe MIS-C (*n* = 25)	*P* value
Constitutional symptoms					
Headache	23 (18.9)	1 (5.9)	10 (12.8)	12 (48.0)	0.000
Myalgia	24 (20.3)	3 (17.6)	18 (23.4)	3 (12.5)	0.49
Dermatologic					
Conjunctivitis	51 (41.8)	7 (41.2)	34 (42.5)	10 (40.0)	0.97
Skin rash	59 (49.6)	9 (52.9)	40 (51.3)	10 (41.7)	0.61
Mucus membrane involvement	18 (15.1)	3 (17.6)	11 (14.1)	4 (16.7)	0.9
Respiratory					
Rhinorrhea	3 (2.5)	1 (5.9)	2 (2.6)	0 (0)	0.49
Cough	35 (29.2)	4 (23.5)	21 (26.9)	10 (40)	0.39
Chest pain	4 (3.3)	0 (0)	1 (1.0)	3 (12.0)	0.021
Tachypnea	28 (23.0)	0 (0)	14 (14.4)	14 (56.0)	0.000
Respiratory distress	25 (20.8)	0 (0)	11 (14.1)	14 (56.0)	0.000
Gastrointestinal					
Hepatomegaly	7 (6.2)	0 (0)	5 (6.7)	2 (9.1)	0.496
Splenomegaly	9 (8.0)	0 (0)	7 (9.3)	2 (9.1)	0.446
Ascites	11 (9.6)	3 (18.8)	6 (8)	2 (8.7)	0.41
Abdominal pain	56 (46.7)	9 (52.9)	35 (44.3)	12 (50)	0.76
Nausea and vomiting	65 (53.3)	5 (29.4)	45 (56.3)	15 (60.0)	0.082
Diarrhoea	44 (36.7)	5 (29.4)	33 (41.3)	6 (26.1)	0.33
Ileocolitis	5 (4.3)	0 (0)	4 (5.3)	1 (4.3)	0.64
Colitis	2 (1.8)	0 (0)	2 (2.7)	0 (0)	0.6
Lymphadenopathy	6 (5)	1 (5.9)	3 (3.8)	2 (8.0)	0.688
Sore throat	7 (5.8)	0 (0)	4 (5.1)	3 (12.0)	0.236
Edema	25 (21.0)	6 (35.3)	14 (19.7)	5 (20.8)	0.282

We also studied some laboratory parameters in our patients including complete blood count (CBC), biochemistry, liver tests (aspartate transaminase (AST) and alanine transaminase (ALT)), prothrombin time (PT), thromboplastin time (PTT), albumin, inflammatory markers (erythrocyte sedimentation rate (ESR), C-reactive protein (CRP)), Ferritin, IL-6, Fibrinogen, D-dimer, cardiac and other enzymes (Creatine phosphokinase (CPK), CPK-MB, troponin, Lactate dehydrogenase (LDH), amylase, lipase), triglyceride and vitamin D level. We found that vitamin D levels in children with severe MISC were significantly lower than children with mild to moderate MIS-C (*P* = 0.05). Table 3 shows laboratory information in details.

**Table 3.** Laboratory findings of the patients with mild to moderate and severe MIS-C

Laboratory characteristics Median (IQR 25–75)		Disease severity		
		Mild and moderate MIS-C	Severe MIS-C	*P* value
CBC	WBC ( × 10⁹ cells per L)	8.350 (6.198–12.178)	10.11 (7.255–15.780)	0.163
	Neutrophils ( × 10⁹ cells per L)	5.700 (3.190–8.035)	8.230 (4.625–12.25)	0.370
	Lymphocyte ( × 10⁹ cells per L)	1.520 (0.985–3.105)	1.400 (0.665–1.920)	0.178
	Hemoglobin (g/dl)	11.4 (10.4–12.6)	11.6 (10.6–12.8)	0.645
	Platelet ( × 10⁹ cells per L)	235 (129–311)	182 (113–247)	0.178
Biochemistry	BUN (mg/dl)	11 (8–15)	15 (11–19)	0.002
	Creatinine (mg/dl)	0.6 (0.5–0.7)	0.7 (0.5–0.8)	0.160
	Calcium (mg/dl)	8.6 (8.1–9.2)	8.4 (7.7–8.9)	0.816
	Phosphorus (mg/dl)	3.9 (3.0–4.7)	3.7 (2.9–4.3)	1.000
	Magnesium (mg/dl)	2.1 (1.9–2.3)	2.0 (1.7–2.2)	0.397
	Sodium (mEq/l)	134 (130–138)	134 (130–1137)	0.889
	Potassium (mEq/l)	3.9 (3.6–4.3)	3.9 (3.6–4.3)	0.831
Liver tests	AST (U/L)	31 (22–59)	41 (26–101)	0.233
	ALT (U/L)	24 (14–51)	27 (16–74)	0.711
	PT (sec)	14 (13–16)	15 (13–16)	1.000
	PTT (sec)	36 (32–40)	34 (31–39)	0.722
Inflammatory markers	Albumin (g/dl)	4.1 (3.6–4.4)	3.3 (2.9–3.6)	<0.0001
	ESR (mm/h)	27 (12–49)	30 (15–53)	0.493
	CRP (mg/l)	28 (6–54)	57 (24–119)	0.346
	Ferritin (ng/ml)	193 (95–441)	457 (170–1462)	0.428
	Interleukin-6 (pg/ml)	76 (11–184)	3 (0- 3.3)	0.167
	Fibrinogen (mg/dl)	377 (255–508)	417 (254–541)	0.379
	D-dimer (ng/ml)	2.8 (0.8–316)	4.2 (1.0–420.2)	0.765
Enzymes	CPK (U/l)	55 (36–105)	72 (31–337)	0.458
	CPK-MB (*μ*g/l)	16 (10–20)	20 (11–37)	0.778
	Troponin	0.1 (0.09–0.1)	0.1 (0.1–4.2)	0.294
	LDH (U/l)	537 (436–670)	558 (416–855)	0.780
	Amylase (U/l)	65 (28–232)	43 (6–117)	1.000
	Lipase (U/l)	32 (20–266)	13 (3–25)	0.598
Others	Vitamin D (ng/ml)	20.5 (8.2–33.7)	8.5 (3.0–14.2)	0.05
	Triglyceride (mg/dl)	192 (132–283)	92 (142–142)	1.000

WBC, White blood cell count; ALT, Alanine aminotransferase; AST, Aspartate aminotransferase; CRP, C-reactive protein; ESR, Erythrocyte sedimentation rate; PT, Prothrombin time; PTT, Partial thromboplastin time; CPK, Creatine phosphokinase; LDH, Lactate dehydrogenase.

We also studied clinical manifestation in the course of the disease. Cardiac involvement was among one of the considerable organ involvements among patients. The most common cardiac presentation was coronary arteries dilation which was detected in 42 (34.4) patients (41.2% (*n* = 7) of the MIS-C group overlapping with KD, 33.8% (*n* = 27) in MIS-C group without overlap with KD, and in 32% (*n* = 8) of cases with severe MIS-C (*P* = 0.809), respectively). Hypotension, myocarditis and valvulitis were also seen. Hypotension and myocarditis were significantly more common among patients with severe MIS-C (*P* < 0.000 and *P* = 0.041, respectively), while valvulitis was only seen in 2 patients with mild and moderate disease (*P* = 1.000). Renal involvement was seen in 4 patients, and was significantly more common among patients with severe MIS-C (*P* = 0.027). In the course of admission, 25 (20.5%) patients required supplemental oxygen, 11 (11.3%) patients with mild and moderate MIS-C and 14 (56.0%) patients with severe MIS-C. This difference was statistically significant (*P* < 0.0001). Twenty-five (20.5) patients experienced PICU admission and 2 (8.0) patients were intubated. All of these patients had severe MIS-C. In contrast, no patients with mild and moderate disease required PICU care or intubation. These differences were significant (*P* < 0.0001for both).

Unfortunately, 2 (1.6%) of 122 patients died and both had severe MIS-C. The mortality rate was 8% among patients with severe MIS-C, while it was not found in cases with mild and moderate disease. As a result, severe MIS-C significantly increased mortality among patients compared to mild and moderate disease (*P* = 0.041). Details are shown in Table 3. The median duration of hospital stay in mild and moderate disease was 6 days and in severe disease was 11 days. This finding was also significant (*P* = 0.000).

Given the current national shortage of IVIG in Iran and effectiveness of corticosteroid based on literature, we used pulse glucocorticoids for patients with moderate or severe illness and high-dose glucocorticoids for patients with mild illness. Corticosteroid was used to treat 92 patients (77.3%); 14 (82.4%) MIS-C subjects overlapping with KD, 57 (74.0%) cases without overlap with KD, and 21 (84.0%) subjects with severe disease (*P* = 0.507). We observed clinical improvement in the way of symptom resolution with the use of steroids in our population.

IVIG was also required in 20 patients (18.3%). Totally, 45.5% of patients with severe disease required this treatment, while it was only prescribed in 2 (12.5%) MIS-C subjects overlapping with KD and 8 (11.3%) cases without overlap with KD, respectively (*P* = 0.001).

Table 4 shows the clinical characteristic in the course of the disease and also the applied management and final outcome of the patients.

**Table 4.** Clinical characteristic in the course of the disease, management and outcome

Disease		Severity variable^a^			
		Total (122)	Mild and Moderate MIS-C (97)	Severe MIS-C (25)	*P*-value
ICU Admission		25 (20.5)	0 (0)	25 (100.0)	0.000
Respiratory	O2 supplement	25 (20.5)	11 (11.3)	14 (56.0)	0.000
	Non-invasive mechanical ventilation	7 (5.7)	1 (1.0)	6 (24.0)	0.000
	Intubation	2 (1.6)	0 (0)	2 (8.0)	0.041
Cardiac	Hypotension	25 (20.5%)	11 (11.3)	14 (56.0)	0.000
	Myocarditis	2 (1.6)	0 (0)	2 (8)	0.041
	Valvulitis	2 (1.6)	2 (2.1)	0 (0)	1.000
	Coronary Arteries dilation	42 (34.4)	34 (35.1)	8 (32)	0.818
Renal involvement		4 (3.3)	1 (1.0)	3 (12)	0.027
Final outcome	Hospital Stay in survived patients (day).	6 (5–10)	6 (4–8)	11 (8–13)	0.000
	Death	2 (1.6)	0 (0)	2 (8.0)	0.041

a all of the values are in Number (%), except for Hospital stay which is median (IQ 25–75).

## Discussion

Herein, we studied the clinical and laboratory characteristic of one hundred and twenty-two patients admitted to our hospital with the confirmed diagnosis of MIS-C. As mentioned before, our first experience of 45 patients with MIS-C has published earlier, on August 2020 [16] and this manuscript is an update of our centre. Furthermore, in the current study, we categorised patients based on disease severity into two subgroups and we compared them with each other: mild to moderate MIS-C and severe MIS-C.

With the exception of fever, which is the cornerstone of the diagnosis, the most observed signs and symptoms at presentation were nausea and vomiting (53.3%), skin rash (49.6%), abdominal pain (46.7%), conjunctivitis (41.8%) and diarrhoea (36.7%). It is almost equal to our previous findings. On the other hand, rhinorrhea, chest pain, hepatomegaly, splenomegaly, ascites, ileitis, colitis, lymphadenopathy and sore throat were seen infrequently, each of them in less than 10% of patients. Among them, headache, chest pain, tachypnea and respiratory distress were significantly more common in patients with severe MIS-C.

In a systematic review conducted by Radia *et al.*, gastrointestinal symptoms were the most common clinical presentation (71%), including abdominal pain, diarrhoea and vomiting; 42% of studied patients suffered from skin rash and cough. They reported respiratory tract symptoms as the least observed manifestations (cough and sore throat) [19]. In a case series of 58 children with MIS-C from 8 hospitals in England, abdominal pain, diarrhoea, rash, shock, vomiting and conjunctivitis were frequently seen (more than 40%). Lymphadenopathy, swollen extremities, sore throat and confusion were the least common clinical features [9].

In a study of MIS-C in Mumbai-India, diarrhoea/vomiting, pain in abdomen, rash and conjunctivitis were observed in more than half of the patients. They also categorised the patients into MIS-C with shock and MIS-C without shock, based on inotrope requirement and/or more than 20cc/kg fluid resuscitation. They found that patients with shock were older than patients without shock [20]; similarly, we found that our patient with severe MIS-C were older than patients with mild to moderate disease. Findings of above studies, about distribution pattern of clinical manifestations at the beginning of the hospitalisation, were almost consistent with our results.

Elevated COVID-19 IgM and IgG were detected in 33.3% and 46.9% of patients respectively. In addition, 36.9% of subjects were SARS-CoV-2 RT-PCR positive. There was no significant difference between two groups in terms of COVID-19 SARS-CoV-2 serology or SARS-CoV-2 RT-PCR. In other studies, the percentage of patients with evidence of past or current COVID-19 infection was reported higher [9, 19, 21].

We studied inflammatory markers and compared them between two groups. ESR, CRP, D-dimer and Ferritin were higher among patients with severe MIS-C, however none of them were statistically significant. Similarly, albumin, as a marker of inflammation, was markedly lower in patients with severe MIS-C. In a systematic review of patients with MIS-C authors found that inflammatory markers were raised in most of the cases, including neutrophilia, elevated CRP level and lymphopenia [19]. In a retrospective case series of children who were hospitalised with the diagnosis of MIS-C in Atlanta, all 11 patients developed hypoalbuminemia and 83% of them received intravenous albumin infusion [22]. In the study of Toreres *et al*. from Santiago de Chile, patients with severe disease had lower albumin and higher D-dimer levels [23]. Our study also confirmed that hypoalbuminemia might be a predictor of severe disease.

Another finding was that vitamin D levels were low in most of the patients and was significantly lower in patients with severe disease. In a review conducted by Feketae *et al*., vitamin D was introduced as a predictor of MIS-C severity. They also made a hypothesis that correction of vitamin D deficiency may possibly improve the clinical course of the disease [24]. The role of vitamin D in COVID-19 has been well studied by several authors previously [25–27].

In our study, 20.5% of patients required ICU admission, all with severe MIS-C. The need for supplemental oxygen, non-invasive ventilation and intubation were also significantly higher in patients with severe MIS-C.

As a consequence of cytokine storm, MIS-C results in multi-organ failure [28]. We found that hypotension and myocarditis were significantly more frequent in patients with severe MIS-C; surprisingly, the rate of coronary artery dilation was almost 35% in each two groups; and it was reported 29% in our previous report. Renal involvement was a rare complication and was markedly more frequent in patient with severe MIS-C. It was in consistent with Pouletty *et al*.'s study. They found that myocarditis found in 86% of patients with severe disease (requiring intensive care) and 55% of patients with non-severe disease; a meaningful finding [29].

In the multi-centre observational study conducted in India, 23 patients with MIS-C were included [20]. They found that LV systolic dysfunction was significantly higher in patients with shock. They also reported that coronary artery dilation was seen in 26% of patients, with no significant difference in patient with or without shock, a finding consistent with our results. In another single centre study done in Philadelphia, Corwin *et al*. defined 3 subgroups for their patients with MIS-C: patient with critical, moderate and mild illness. They found that coronary artery dilation was seen only in 2 of 5 patients with severe illness and none of patients with moderate and mild disease; nonetheless this finding was not statistically significant [30]. So, we can conclude that coronary artery dilation is a relatively common finding in patients with MIS-C, regardless of disease severity. Although there is no clear aetiology for coronary artery dilation, it can be secondary to vasculitis or systemic hyperinflammation, and this abnormality usually resolves within 30 days [31].

Unfortunately, 2 of the patients with severe MIS-C died, while all children with mild to moderate MIS-C survived. In a study from England [9], the rate of intubation was 43%, which is clearly higher in comparison to our patients. They reported that 14% of patients experienced coronary artery aneurysm (35% in our study). The mortality rate of their patients was almost similar to our study (2% in mentioned study *vs* 1.6% in our study). In our first report, the mortality rate was 11% [16]. This decrease may be attributed to the increased experience and publication of reliable guidelines in the management of such a novel syndrome.

In another study from the USA, 28 children with MIS-C were reviewed retrospectively. Sixty-one per cent of cases required ICU admission, 43% received supplemental oxygen and 25% required noninvasive ventilation; acute kidney injury was seen in 21% of patients [21]. All of these complications were higher among their patients compare to ours, however they reported no death. These results might be explained in part by racial difference, better care and more equipped facilities in a developed country.

The initial steps in the management of critically ill children with COVID-19 are supportive cares, including oxygen supplement and hemodynamic support. The goals of treatment are decreasing systemic inflammation in order to lessen morbidity and mortality. For these purposes, IVIG, corticosteroid and tocilizumab have been suggested by authors [21, 30]. In our centre, IVIG and corticosteroid are commonly prescribed. Totally, corticosteroid and IVIG was prescribed in 77.3% and 18.3% of our patients respectively and IVIG was significantly used more frequently in patients with severe disease. In other studies, IVIG was prescribed clearly more frequently; it can be well explained by the fact that IVIG is among the scarce drugs in Iran, mainly due to high cost and insurance policies; so, the prescription is completely strict and only in definite indications with approved efficacy.

To our knowledge, we reported a relatively large numbers of MIS-C. However, it has some limitations. Our centre is a referral hospital and several children with complex complicated diseases have been referred to our hospital. Our study may therefore represent a more severe form of disease, and the results should be interpreted with caution.

## Conclusion

In conclusion, patients with MIS-C in our region suffer from wide range of signs and symptoms. Among laboratory parameters, hypoalbuminemia and low vitamin D levels may predict a more severe course of the disease. Coronary artery dilation is frequently seen among all patients, regardless of disease severity. Mortality rate was 1.6% in our study, all in patients with severe disease.

## Data Availability

The patient data and datasets used and/or analysed during the current study are available from the corresponding author on reasonable request.
